# New Advances in the Diagnosis and Treatment of Mental Disorders

**DOI:** 10.3390/diagnostics14212378

**Published:** 2024-10-25

**Authors:** Diogo Telles-Correia

**Affiliations:** 1 Clínica Universitária de Psiquiatria e Psicologia Médica, Faculdade de Medicina, Universidade de Lisboa, Av. Prof. Egas Moniz MB, 1649-028 Lisboa, Portugal; tellesdiogo@gmail.com; 2 PSYLAB-Instituto de Saúde Ambiental, Faculdade de Medicina, Universidade de Lisboa, Av. Prof. Egas Moniz MB, 1649-028 Lisboa, Portugal

One of the fundamental aspects of research in psychiatry, and what makes it such a complex area, is its methodological specificities. Without taking these specificities into account, the research results are null and of little use [1,2].

Mental disorders, like medical disorders in general, are situations that cause biological (death or risk of death) or psychosocial (distress and disability) harm to those who suffer from them, and which can, through tested methods (biological, psychotherapeutic) be attenuated or even eliminated [3,4,5].

However, there are some specificities in psychiatry that must be considered. These range from its models of causality (how neurobiological components and psychosocial variables contribute and interact) to the concepts themselves that are involved in this area (what a mental disorder is and what it means, what the classification of mental disorder is and what it means, etc.).

For this reason, research methods (epistemology) in psychiatry must differ, in some aspects, from those used for the study of medical disorders in general. They have specificities. Models imported directly from the rest of medicine, mainly based on methods used in natural sciences, cannot be fully replicated [1].

Regarding causality, there is increasing evidence that the factors that intervene in the causality of psychiatric disorders interact in a complex way and belong to different paradigms and levels of abstraction [6,7,8].

It is well established that psychosocial factors such as life experiences have a strong causal relationship with psychiatric illnesses. These should be explored through a different language that does not belong to natural sciences (ex neurosciences), but to social and human sciences [9].

On the other hand, biological factors may not act directly on psychiatric disorders but interact in a complex way together with psychosocial variables (e.g., life experiences, sociocultural environment). Genes can also have an indirect influence on the causality of certain psychiatric illnesses. For example, those that predispose individuals to a certain temperament or personality, which in turn can predispose individuals to depression [10,11].

Certain mental states, with complex causality, can also influence the environment and on other mental states. For example, a person with a personality prone to greater impulsivity may cause a more adverse relational environment, which in turn may predispose them to depression [5].

Culture, and the type of society where the person grows up or lives, may act as a determining factor in the onset of mental symptoms or mental disorders. For example, a society that devalues certain types of behavior or characteristics of a person, may favor the emergence of symptoms or mental disorders in that person [12,13,14,15].

Another essential feature is the tendency to accept that there is a continuity between mental states considered normal and the ones that are abnormal. What distinguishes a normal mental state from a non-normal one is not a biological variable, but a clinical assessment (values-dependent) [16,17].

In neurosciences, what we can find are the neurobiological correlates of these clinical situations (which cause distress/disability to patients). This does not mean that the distinction between normal and pathological in psychiatry is made through neurobiological markers [1].

Thus, biological correlates can be discovered for the behaviors considered disturbed and these conclusions can lead to the discovery of new drugs that can improve these conditions. But these should only be used in situations that are considered pathological by non-biological factors, as explained above.

In recent times, a critical look has been taken at the methods used in the diagnosis and research carried out in this area [18] and some more original strategies have been developed to overcome these issues [19,20].

It is essential that new ways of critical thinking and researching continue to be developed, to improve the results achieved in this area.
